# Analysis of the Accuracy of Mass Difference-Based Measurement of Dry Clutch Friction Material Wear

**DOI:** 10.3390/ma14185356

**Published:** 2021-09-16

**Authors:** Matija Hoić, Alen Miklik, Milan Kostelac, Joško Deur, Andreas Tissot

**Affiliations:** 1 Faculty of Mechanical Engineering and Naval Architecture Ivana Lučića 5, University of Zagreb, HR-10002 Zagreb, Croatia; alen.miklik@gmail.com (A.M.); milan.kostelac@fsb.hr (M.K.); josko.deur@fsb.hr (J.D.); 2 Ford-Werke GmbH, 50769 Cologne, Germany; atissot1@ford.com

**Keywords:** dry clutch, experimental characterization, moisture influence, measurement, testing, wear

## Abstract

The paper demonstrates that the dry clutch friction plate wear rate, measured based on the plate mass difference method, exhibits a transient behavior after each change of friction interface temperature level. The effect is hypothesized to be caused by a temperature-dependent change in the moisture content/mass level in the friction material. To test this hypothesis, a series of synchronized characterization experiments have been conducted by using two friction plates, one for wear tests and the other for drying in an oven under the same temperature conditions. Based on the analysis of test results, a moisture content compensation procedure, which reduces the transient wear rate from being 100% to being 50% higher compared to stabilized wear rate, is proposed and verified. The gained insights are used to set recommendations on the organization of routine wear characterization experiments aimed at avoiding the effect of moisture content influence on the accuracy of wear measurement. The main recommendations are to minimize the number of temperature target level changes through proper design of the experiment, insert a run-in test after every long test pause, and execute a pre-heat, blind wear test at the beginning of each test day.

## 1. Introduction

Dry clutches have recently been considered for implementation in different hybrid [1,2] and fully electric [3] vehicles, in addition to being used in manual transmissions, automated transmissions, such as AMTs and DCTs [4,5], and E-clutch-based transmissions [6]. Accurate clutch models are required for simulation, monitoring, and control purposes of the above-mentioned transmissions. Two features of clutch friction material, which have significant implications on transmission design and control, are coefficient of friction (COF) [7,8] and wear [9]. Due to complex physical backgrounds, both are typically modeled based on experimental characterization results [7,8].

The term dry wear relates to several types of interactions between two sliding bodies and subsequent loss of material [10,11,12]. Based on the underlying physical processes, dry wear is typically described as either adhesive or abrasive wear and reported to occur in dry clutches, brakes, non-lubricated plain bearings, etc. [10]. A practical approach to modeling of wear typically relies on the application of Archard’s law of wear [13], which defines the wear rate parameter (*w*) as a quotient of the worn volume *V_w_* and the friction-interface dissipated energy *E_diss_*:

*w* = *V*_*w*_/*E*_*diss*_. (1)

Various experimental characterization studies have shown that the wear rate for clutch and brake friction materials is affected primarily by temperature [13,14,15], but it can also be influenced by slip speed [11,16,17], normal load [11,18], and closing time [17]. Furthermore, some specifics of operating conditions and parameters can affect the wear process, such as the occurrence of wear debris [19], the shape of friction plate grooves aimed at the removal of particles, increased variability of wear beyond the end grooves [20], and a relatively sharp wear transient from high to regular values during the run-in phase [21,22].

Therefore, realistic characterization tests aimed at producing an accurate wear model should involve the full friction plate sliding over a disc cut from the real clutch pressure plate or flywheel. In other words, a full-size disc-on-disc tribometer should be used rather than a pin-on-disc tribometer. Such a tribometer has been designed in [23] and used to parameterize a wear model, which consists of static wear rate dependency on three operating parameters (temperature, slip speed, and closing time) and a run-in transient sub-model [17].

Equation (1) suggests that the characterization of wear rate involves the measurement of the difference in worn volume for a given increment of dissipated energy. As described in [23], when the full dry clutch friction plate is used as a sample for realistic wear characterization, two distinct approaches of determining the difference in volume can be used. The first approach relies on measuring the difference in thickness (Δ*d*) and the known friction plate surface (*A_fr_*):

*V*_*w*_ = *A*_*fr*_Δ*d*. (2)

The second approach is based on sensing the difference in mass (Δ*m*) and the known density of friction material (*ρ*):

*V_w_* = Δ*m*/*ρ.* (3)

It is shown in [23] that the second, mass-difference-based approach yields more reliable results for test procedures based on small worn volume increments, which are needed to characterize wear for a large set of operating parameters in a reasonable time frame [23]. This is because the thickness measurement is sensitive to thermal expansion effects and piece-to-piece variation of the friction plate woven-spring compliance.

However, the mass measurement accuracy can be sensitive to the moisture content in the friction material. This is because of the hydroscopic properties of the organic compound of the composite friction materials of dry clutches, which can appear in the friction material as both a matrix and binder (phenolic resin and palm fibers, [24]), matrix and portion of the fiber (phenolic resin and copper/cellulose fiber, [25]), or solely for the matrix (unsaturated polyester [26]). The moisture content depends on the ambient temperature and relative humidity. Although different levels of ambient temperature and humidity are reported in different experimental studies (20–27 °C and 62–70% in [27], 26 °C and 58–62% in [28], room temperature and 45–55% in [29], 21 °C and 45% in [30], and 20–24 °C and 45–55% in [31]), commonly they are tried to be kept constant. In addition, other conditions, such as the rig operating temperature and duration and friction plate storage conditions related to the periods between removing the plate from the rig to conducting the mass measurement, may also affect the moisture content. The moisture content is typically tried to be regulated by drying the friction material at temperatures up to 100 °C before the mass measurement [32,33]. Further, the friction material is stored in a de-humified container, and the humidity variation is mitigated by keeping the lab at a constant room temperature at all times. Finally, it should be taken into account that the wear rate can be affected by the aging of the friction material [34,35]. Therefore, the characterization tests should involve friction plates from the same production batch.

In this paper, an experimental analysis of the possible influence of a change of the three aforementioned operating parameters on the wear rate transient behavior has first been presented. It is demonstrated that the wear rate transient occurs only with respect to temperature, and the effect is presumably contributed to the influence of moisture content variation. Next, an experimental analysis involving synchronized tests of two friction plates is conducted, where the first plate is subject to wear tests on the rig, and the second one is subject to drying in an oven under the same temperature conditions as on the rig. The test results are used to check the hypothesis of moisture influence on wear characterization, based on designing and applying a procedure of compensating the raw wear rate values based on the dried plate mass difference measurement results. The synchronized tests involving two plates at a time should be avoided in routine tests, as they are time- and resource-consuming. Therefore, the insights gained through the analysis are employed to recommend the proper organization of single-plate wear tests to mitigate the moisture content variation influence.

The main contributions of the presented study are: (i) experimental analysis of air moisture influence on the accuracy of the dry clutch friction plate, mass difference-based wear rate measurement, and (ii) recommendation of wear test organization to mitigate the moisture influence.

## 2. Disc-on-Disc Tribometer

**Figure 1:**
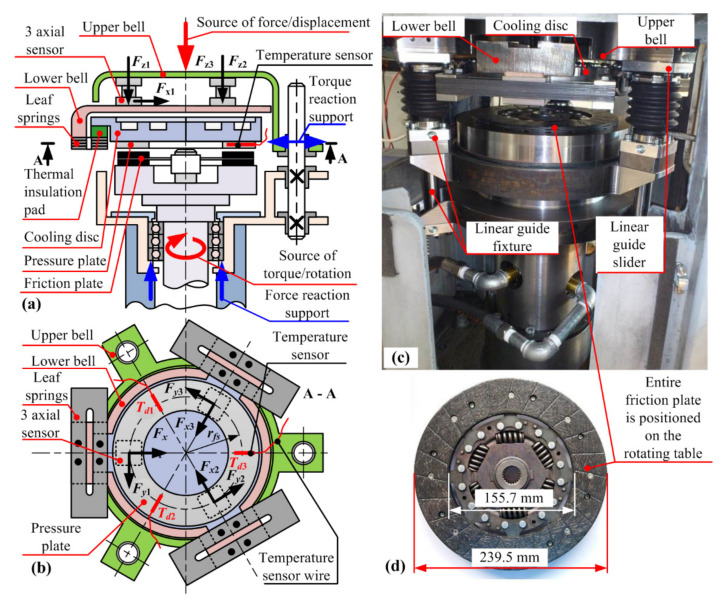
Functional schematics (**a**,**b**) and photographs of the redesigned disc-on-disc tribometer rig (**c**) and friction plate (**d**).

The disc-on-disc-type CNC tribometer (Figure 1, [17,23,36]) has been developed for the purpose of conducting characterization tests of dry clutch friction coefficient and wear over a wide range of operating parameters and different friction materials while using an entire friction plate as a sample. The non-rotating disc (i.e., the pressure plate) is attached to a source of variable normal/axial force (vertical axis), while the rotating disc (i.e., the friction plate) is placed on a rotating table connected to a source of variable torque (rotational axis). The pressure plate is connected to the vertical axis by utilizing a set of three precise, stiff, and compact 3axial force piezoelectric sensors; thus, preventing any parallel (typically friction loss) force/torque transfer paths that would affect the measurement accuracy. A custom-designed leaf spring-based suspension system of the vertical axis is designed to ensure a uniform contact over the single friction pair of bodies (pressure plate against the single friction plate surface) and provide torque transfer from the pressure plate towards the normal force/torque sensor.

The arrangement in which a single friction surface is used, unlike in an actual clutch where both sides of friction plate are in contact, simplifies the tribometer design. The arrangement with rotating friction plate results in simplified pressure plate temperature sensing and water cooling, and it enables centrifugal forces to remove the worn particles from the friction plate during the acceleration phase when the clutch is open. More details on the tribometer mechanical, measurement, and control system design, as well as the organization of experiments, can be found in [23].

When compared to the original tribometer design from [17,23], the upgraded tribometer design described in [36] and concerned herein includes the following enhancements aimed at increasing the system natural frequency and substantially reducing sensitivity to shudder vibrations (Figure 1): (i) setting of three circumferentially-distanced linear guides, (ii) reducing the length of bending force cantilever through placing the linear guides and the leaf springs in the same horizontal plane with the friction contact surface, (iii) applying three circumferentially-distanced three-axial piezoelectric force sensors instead of a single, two-axis force and torque sensor, (iv) reducing the cooling disc radius and thickness, and (v) redesign of the thermal insulation element by means of reducing contact surfaces between the cooling disc and the vertical axis.

## 3. Basic Specifics of Friction Pair Materials

**Figure 2:**
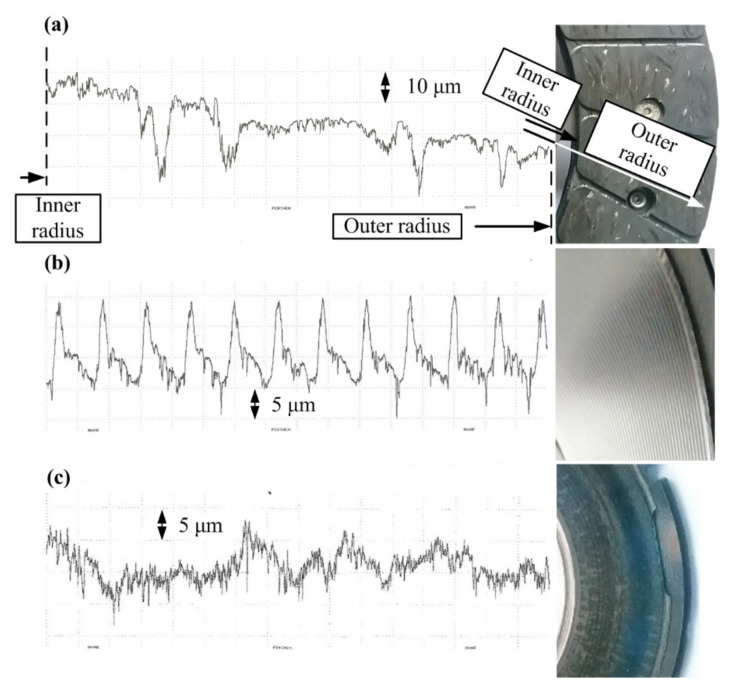
Surface roughness of friction plate (**a**), and unused (**b**) and worn-down pressure plate (**c**).

The friction plates considered (see Figure 1d and Figure 2a for zoom-in detail) are used in European B- and C-segment passenger vehicles. It is made of a composite friction material for which the Scanning Electron Microscope (SEM)/Energy Dispersive Spectroscopy (EDS) analysis, conducted in accordance with ASTM E1508 standard, has shown that it is composed of an organic matrix reinforced with fiberglass threads and copper-based metal wires. Each friction disc includes radial grooves (Figure 1d), which facilitate the removal of regular friction material particles during sliding conditions. Two opposing friction discs are riveted onto wave springs, which improve contact surface alignment. The wave springs are connected to the central splined hub via torsional dampers. When tested on the tribometer rig, the friction disc is worn against a pressure plate (Figure 1a,b), which is represented by an 8 mm thick grey cast disc made by machining the clutch flywheel while keeping the contact surface in its original condition (as shown Figure 2b).

Figure 2 includes characteristic surface roughness plots for friction and pressure plates. The friction material (Figure 2a) exhibits a rather smooth surface, with impressions that correlate with the positions of the copper wires. The overall surface appears to be tilted towards the outer edge. The new pressure plate (Figure 2b) exhibits a leveled overall surface with clearly distinguishable peaks, which correspond to the circular protrusions visible on the contact surface. A fully worn-down pressure plate (Figure 2c) exhibits a smoother surface, as a consequence of a large number of recorded experiments, during which 20 friction plates were worn down against this single pressure plate.

## 4. Organization of Experiments

A standardized test procedure and related apparatus have not been developed for dry clutches, unlike for wet clutches, which are typically characterized in accordance with the SAE #2 Friction Test Rig recommendations [37]. SAE #2 machines operate by considering the clutch as a brake to bring a tunable-inertia flywheel from a predetermined initial speed to a full stop. Similarly, the organization of experiments considered herein is based on the goal of mimicking the regular dry clutch operation with respect to both clutch actuation sequence and range of operating parameters. Thus, both the specimen geometry and the organization of experiments support the conditions of the wear process as in the real vehicle.

**Figure 3:**
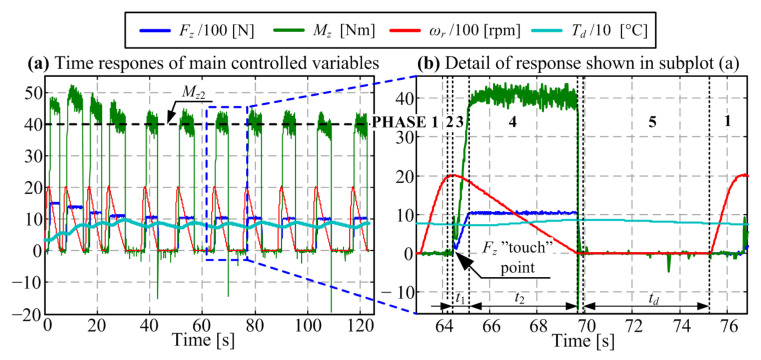
Experimental responses illustrating the organization of experiments (particular operating point corresponds to initial slip speed *ω_r_*_0_ = 2000 rpm, torque *M_z_*_2_ = 40 Nm, and closing time *t*_2_ = 5 s).

Each experiment consists of a large number of clutch closing cycles (Figure 3) under a given combination of model input parameters (temperature, slip speed, torque, and closing time). Each test cycle consists of the following phases (see Figure 3b): (1) ramping up the slip speed *ω_r_* to the target level (*ω_r_*_0_) under idling operation (no clutch friction load), (2) bottoming the vertical axis, (3) ramping up the normal force *F_z_* (interval *t*_1_), (4) keeping the normal force at the target level *F_z_*_2_ (related to target torque level *M_z_*_2_) until the slip speed drops to zero (interval *t*_2_), and (5) lifting up the vertical axis and imposing a cooling delay needed to control the friction interface temperature at the target level (interval *t_d_*).

According to Equations (1) and (3), the friction material wear rate is determined based on the difference in friction plate mass (Δ*m*) measured before and after each wear characterization experiment, and the energy dissipated (*E_diss_*) during the experiment. The weight measurement was conducted by means of a scale by Mettler Toledo, model JE3002GE (3200 g capacity, 0.01 g readability). Due to hygroscopic features of the friction material, the air moisture content may considerably affect the results. To mitigate the moisture influence, the following operational procedure was applied: (i) the friction plate was stored in a closed box with silica gel bags whenever it was not used in testing, and (ii) the friction plate was dried in an oven at 100 °C for 1 h at the beginning of each workday, and the weight was measured before and after the drying procedure. The post-drying weight measurement was used for wear rate reconstruction.

The dissipated energy is calculated online (by tribometer control software) as:

*E*_*diss*_ = Σ *M*_*zi*_ ω_*ri*_
*T*_*s*_ (4)

where *M_zi_* and *ω*_ri_ are the actual values of friction torque and slip speed sampled at the rate of *T_s_* = 2 ms. The torque is determined from the tangential force readings of the three-axial sensors (*F_y_*_1_, *F_y_*_2_, *F_y_*_3_) multiplied by the sensor placement radius with respect to the main vertical axis (*r_fs_*; see Figure 1):

*M_z_* = (*F_y_*_1_ + *F_y_*_2_ + *F_y_*_3_) *r_fs_* (5)

For more details on the organization of experiments and wear measurement procedures, the interested reader is referred to [17,23].

## 5. Wear Rate Behavior with Respect to Change of Operating Point

After the end of the run-in phase for a new friction plate (recorded as described in [17]), a set of wear characterization experiments was recorded, where a single operating parameter (slip speed, torque, and temperature) was changed in a stepwise manner, followed by a wear rate stabilization interval, new change of operating parameter, etc. At the start of each day, which correlated with the operating parameter change, drying and mass measurement was conducted, as described in Section 4. In addition, the mass of the hot friction plate was measured immediately after each run, consisting of 250 clutch closing cycles to capture potential transient behaviors [23]. The “hot” mass measurements after *n* × 250 cycles, where *n* ≥ 3 is higher for lower wear rate operating points, are used to calculate the mass difference, the worn volume, and finally, the wear rate.

**Figure 4:**
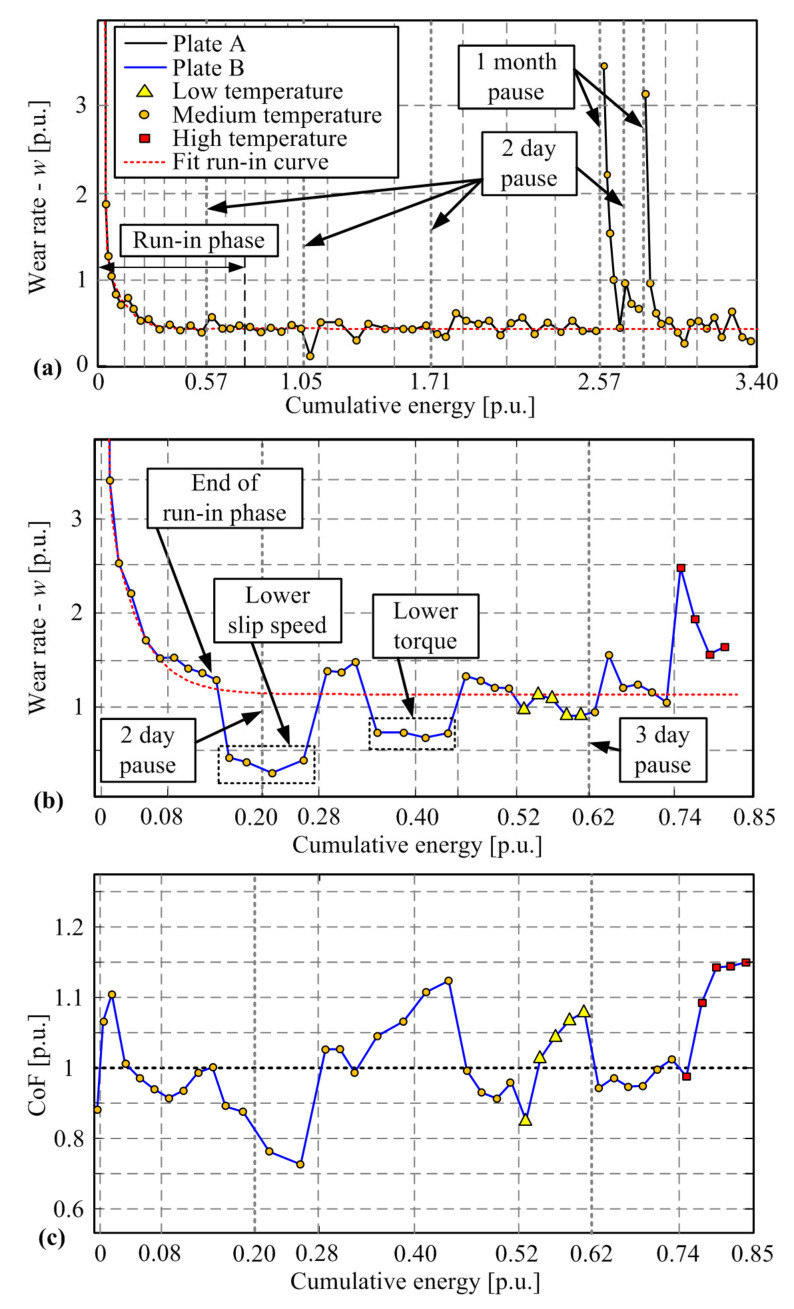
Long-run-in characterization under lower slip speed conditions for Plate A (**a**), and wear rate (**b**) and COF response (**c**) with respect to change of slip speed, torque, and temperature level for Plate B.

The wear characterization experiments are typically conducted day after day, except for weekends or holidays. Figure 4a shows a long-experiment wear rate response, which, apart from regular daily tests, includes weekend and long holiday (approx. 30 days) pauses. This response and further results are given in a normalized (per unit, p.u.) form, where the wear rate output is normalized with respect to average stabilized wear rate for a group of plates considered in [6] and run-in/run-out operating parameters set. Similarly, the dissipated energy is normalized with respect to cumulative energy required to wear down a single friction disc (close to the groove-edge boundary) under the combination of operating parameters, for which the standard run-in and stabilized wear/run-out tests are recorded. The temperature range is such that the temperatures designated as Low and High in Figure 4 correspond to around 100 and 250 °C, respectively.

Figure 4a indicates that immediately after the weekend pause, the wear rate can noticeably differ from the stabilized wear rate level obtained for a given, constant set of parameters. However, the subsequent wear rate point is already close to the expected/stabilized level. After the long holiday pause, the wear rate substantially increases and exhibits a gradual, run-in-like transient towards the stabilized value. It is reasoned that the weekend pause-caused wear rate change is predominantly influenced by the absorbed moisture, which increases the mass of the friction plate, thus typically resulting in smaller mass difference and lower wear rate when compared to the stabilized level. The substantially increased wear rate behavior after the long-holiday pause is presumably related to friction plate surface oxidation.

The wear rate response with respect to the change of operating point is shown in Figure 3b. Note that the dissipated energy grid lines correspond to the start of each day, i.e., the moment of change of operating point. Evidently, the change of torque and slip speed levels from the run-in phase values does not induce any noticeable wear rate transient effects. However, the change of temperature level shows a clear occurrence of wear rate transient effects, both during the transitions from minimum to medium temperature level and from medium to maximum temperature level (Figure 4; the effect is more emphasized for the latter condition).

The wear rate peak occurring after the increase of temperature could not be caused by the friction plate surface oxidation effect because the pauses were either absent or short (2–3 days). Note that the temperatures were kept below extremely high temperatures where a fading effect would occur. The fading effect is manifested in a significant drop of coefficient of friction (COF) [38] that is connected with a loss of clutch torque capacity [39,40] and a substantial rise of wear rate [36]. The fading effect has been reported to occur for temperatures in excess of 250 [41], 300 [42], or even above 450 °C [43] depending on friction material, and it is explained by evaporation and/or melting of the organic compound of the friction material [39,40]. Since the considered temperature was below the fading level and the strong COF drop was not observed (Figure 4c), it has been concluded that the observed wear rate transient behavior is not caused by the fading effect, either.

The next hypothesis was that the wear rate transient was caused by a difference in moisture content in the friction material due to temperature change. The transition to a higher temperature level may cause moisture content to decrease, thus resulting in the increase of measured mass difference and consequently in the wear rate peak, as observed in Figure 4b. In addition, one might reason that a permanent loss of material (e.g., resin) could occur due to evaporation when exposed to high temperatures, which would again be manifested as a wear rate increase.

In order to gain insight into the source of mass loss at high operating temperatures, a new set of experiments was arranged, where two friction plates were tested in parallel: the first one to be worn on tribometer at different temperature levels, and the second one to be exposed to the same temperatures but in an oven (no wear). The test results are presented and analyzed in the next section.

## 6. Detailed Characterization of Moisture Influence on Wear Rate Measurement

### 6.1. Plan of Experiments

**Figure 5:**
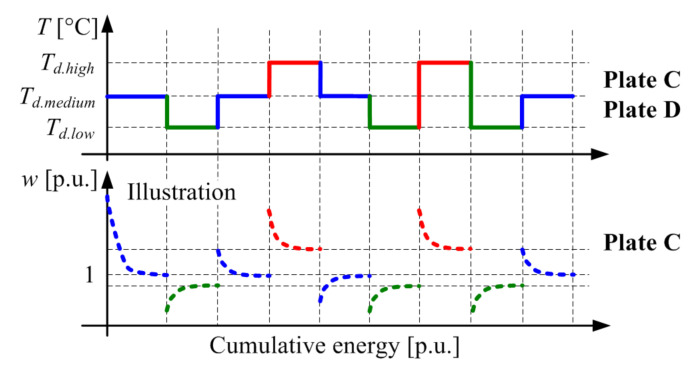
Testing plan related to detailed characterization of moisture influence on wear rate.

Two new plates, designated as Plate C and D, were simultaneously tested according to the test plan illustrated in Figure 5. Plate C was subject to tribometer rig-wear tests, as described in Section 5, but with the temperature being the only parameter changed. Consequently, Plate C experienced the loss of mass due to the wear mechanism and potentially due to temperature increase. Plate D was dried in an oven in parallel with executing the wear tests for Plate C, where the oven-controlled temperature was the same as the target temperature for wear tests. Hence, Plate D was exposed to the same temperature-induced weight loss as Plate C, but without any mechanical wear. When not tested, both plates were being stored in the same box with silica gel bags contained.

It is worth mentioning that apart from being exposed to the same temperature profile, Plates C and D were from the same shipment and were stored together from the end of the manufacturing process to the start of the experiments conducted. The tribometer rig is fully computer- and feedback-controlled, and it has been previously used for long-term experiments related to different aspects of wear characterization [6,17,36]. Hence, the rig is proven to be capable of stable operation for a prolonged period without affecting the wear process (for an illustration of stability of measured wear rate, see the post-run-in response in Figure 4a).

### 6.2. Analysis of the Weight Measurement Results

**Figure 6:**
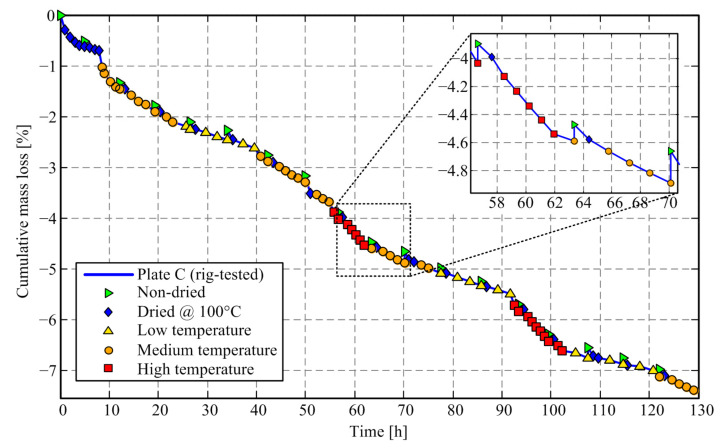
Comparative time response of cumulative mass loss of rig-tested friction plate C.

**Figure 7:**
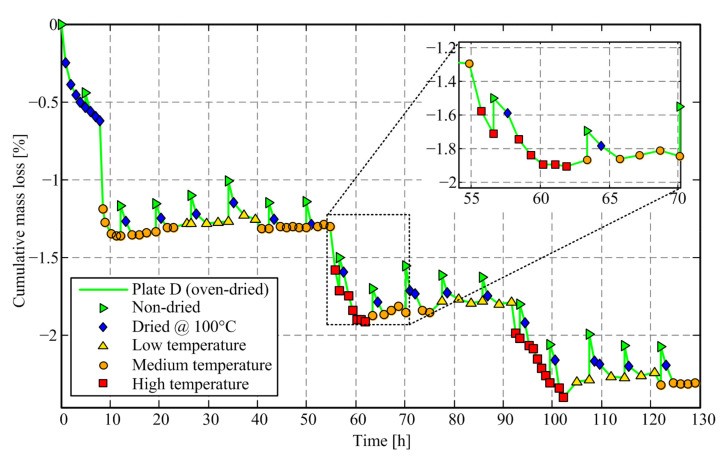
Comparative time response of cumulative mass loss of oven-dried friction plate D.

Comparative time response of cumulative mass loss of friction plates C and D are presented in Figure 6 and Figure 7, respectively. The corresponding numerical results are shown in Table 1. Every new day starts with a non-dried friction plate mass measurement, and it is followed by the initial mass measurement after conducting drying (both plates) in an oven at 100 °C. Unlike in Figure 4, the stepwise change of temperature is set during the workday, i.e., it is always avoided that the change of temperature level coincides with the cold rig conditions.

The results shown in Figure 6 and Figure 7 (see also Table 1) indicate that for the oven-dried Plate D, the mass loss increases with the increase of temperature level, and the mass does not fully recuperate when the temperature returns to the low-temperature level. Strong odor and smoke were observed during drying experiments. The same was not observed for the rig-tested Plate C. Therefore, it may be concluded that a portion of the mass loss during the periods of temperature increase is related to moisture content evaporation; however, a significant amount of lost mass is apparently related to the vaporization of friction material (presumably resin), at least for the oven-dried Plate D.

The details presented in Figure 6 and Figure 7 point to the effect of a significant increase of friction plate mass after an overnight pause, which is present for both dried (Plate D, Figure 7) and worn plates (Plate C, Figure 6) and is explained by moisture absorption. The difference caused by drying at 100 °C is comparable to worn mass during tribometer tests, which illustrates the importance of the initial drying procedure when relying on mass difference measurements when characterizing the wear rate.

The mass loss of Plate C includes contributions of both the mass of worn-down friction material volume and the moisture and eventually resin mass loss due to evaporation when exposed to high temperatures. The mass loss of Plate D is related solely to the moisture and resin evaporation and may, thus, be used to compensate for the same effect of Plate C and determine, more accurate, net wear rate results.

**Figure 8:**
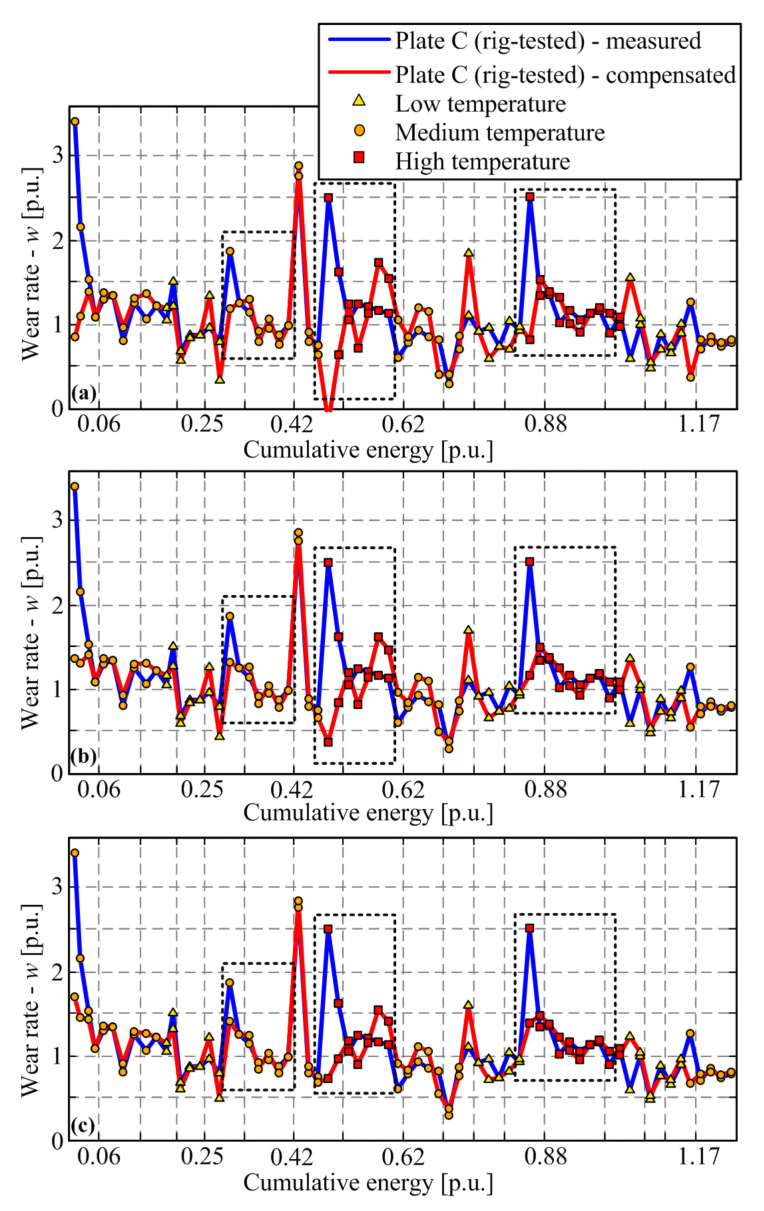
Comparative time responses of mass loss between subsequent wear experiments for friction Plates C and D (derived from Figure 6 and Figure 7, with omitted Non-dried and Drying @ 100 °C points).

Figure 8 shows the corresponding responses of relative mass loss between subsequent wear experiments/measurements. The mass difference points related to drying at the start of the day (Non-dried and Dried@100 °C points from Figure 6) are removed since they are not related to wear. The occurrence of negative mass loss values is explained by the moisture content increase during the overnight pause or due to the transition to a lower temperature level, as well as by a certain influence scale measurement imprecision.

The results in Figure 8 show that, again, the first mass loss after the increase of temperature level (to medium and particularly to high-temperature levels) has a distinctively higher level, i.e., an overshoot, when compared to (most of) the subsequent points for both Plate C and Plate D. It is hypothesized that this mass loss peak is due to removing the moisture content when switching to the higher temperature level. Distinctively high and low wear rate values also occur at the first point in the day, i.e., under the cold-rig conditions.

Figure 8 also shows that the mass loss for high-temperature levels does not drop to zero, which indicates that there are two sources of mass loss during drying experiments (Plate D): one related to the moisture content evaporation, which is dominant in the early stage of mass loss response; and the second one related to the evaporation of the friction plate material itself, presumably its resin component. It is further assumed that the friction material evaporation does not occur on the test rig because there is no evidence for that behavior in the available literature and because the thermal conditions in the oven and on the rig are different: the oven uniformly heats the material, while on the rig the friction interface is hot (hotter than what sensor measures), while there is a large temperature gradient through the material as its thermal conductivity is low [44]. Note that the steady-state value of mass loss response for the rig-tested plate (Plate C) corresponds to the wear process, which is not present in the case of Plate D.

### 6.3. Moisture Content Compensation Procedure

**Figure 9:**
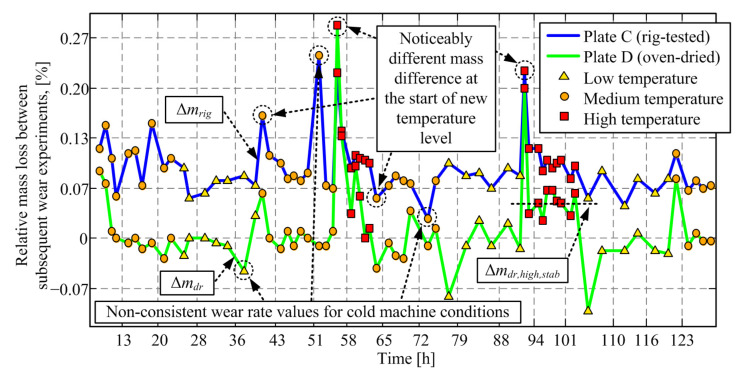
Illustration of compensation of mass loss response for rig-tested plate based on the oven-dried plate response with removed steady-state offset at each temperature.

The proposed compensation procedure is based on correcting the rig-tested plate mass loss in each discrete time instant (Δ*m_rig_*(*i*), blue line in Figure 9) by means of subtracting the moisture content-related mass loss reconstructed from the response of the oven-dried plate. The moisture-related correction is reconstructed by reducing the actual dried plate mass loss at the given temperature (Δ*m_dr,T_*(*i*)) with the stabilized/steady-state mass loss at the same temperature level (Δ*m_dr,T,stab_*), and then dividing the result by the factor of two to account for the fact that only a half of the friction plate is exposed to high temperatures on the rig. Hence, the compensation law reads (see Figure 9 for illustration):

Δ*m*_*comp*_(*i*) = Δ*m*_*rig*_(*i*) − (Δ*m*_*dr,T*_(*i*) − Δ*m*_*dr,T,stab*_) / 2, (6)

The stabilized mass loss of dried plate is subtracted from the transient values because it has been assumed that it represents the mass loss due to friction material resin evaporation, which is present in the oven (Plate D) but not on the rig (Plate C). It can be readily shown that if the stabilized dried plate mass loss were not subtracted, the compensated rig-tested plate mass loss (and thus the wear rate) would not show a distinct increase with temperature. Namely, unrealistic overcompensation would occur, which confirms the assumption on non-existing resin evaporation under the real clutch testing/exploitation conditions. The moisture-related correction, shown in Figure 9 by the green line, has a distinct peak after the temperature increase (particularly when switching to the highest temperature) and diminishes afterward. Applying this correction to the rig-plate measured mass loss response (blue curve) significantly reduces the transient peak in the compensated mass loss response (red curve).

**Figure 10:**
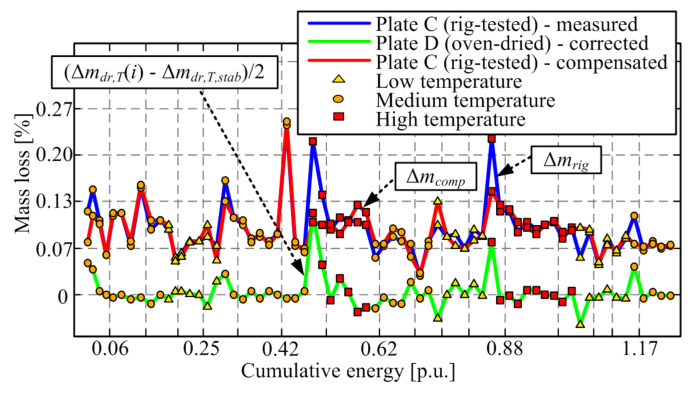
Comparison of moisture content compensated and uncompensated wear rate response for the rig-tested friction plate.

Both uncompensated (Figure 9, blue line) and compensated mass losses (Figure 9, red line) are used to calculate the corresponding wear rates by applying Equations (1) and (3). The resulting compensated wear rate response, shown in Figure 10 by the red line, shows the reduced transient peak when compared with the uncompensated response (blue curve), while the steady-state wear rate consistently grows with temperature. Note that the wear rates are also lowered during the run-in stage of the response. The fact that the transient peak is still present in the majority of compensated response section may mean that (a) there is remaining wear rate dynamics for any other reasons, or (b) the compensation method is not very accurate due to differences between the thermal conditions for oven-dried and rig-tested friction plates. Regarding the latter cause, one may argue that the factor ½ in Equation (6) might be higher because the non-worn side of the friction plate would be affected to some extent by heat dissipation on the worn side or because the friction interface temperature would considerably exceed the measured temperature at a point placed 4 mm beyond the friction interface plane.

**Figure 11:**
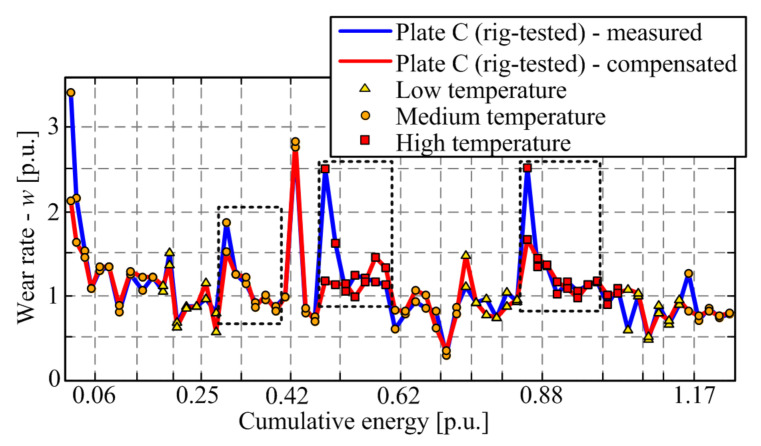
Comparison of non-compensated and compensated wear rate for Plate C when applying factor 1/1 (**a**), 1.25 (**b**) and 1/1.5 (**c**) instead of the “default” value 1/2 in Equation (6).

To examine the above hypothesis, Figure 11 shows the compensation results when increasing the oven-dried plate mass loss scaling factor from its default value of 1/2 (see Equation (6) towards 1. Evidently (and expectedly), the use of factors 1/1 and 1/1.25 gives unrealistic, overcompensated results, including negative wear rate values (Figure 11a,b). Using the factor of 1/1.5 (Figure 11c) yields the results where a large portion of wear rate transient for all response sections may be explained by the moisture influence. However, the compensation slows down the wear rate response in one of the sections (the middle one, i.e., introduces a transient of another type) and makes the run-in response rather shallow and thus possibly unrealistic. Therefore, the compensation factor should be set to (or close to) its default value of ½, as given in Equation (6).

## 7. Conclusions

Possible dry clutch wear rate transient effects related to the change of influential operating parameters have been investigated. While the change of torque and slip speed levels did not result in observable wear rate dynamics, the change of temperature level induced distinct wear rate transients, especially when switching to a high-temperature level. More specifically, the very first wear rate value after the temperature level transition peaked to be approximately 100% higher compared to the stabilized wear rate for the given temperature level. It has been hypothesized that the transient is predominantly caused by the influence of temperature-dependent moisture content dynamics on the accuracy of mass difference-based wear rate measurement.

In order to provide a detailed characterization of the moisture influence on wear rate measurement, two characteristic experiments involving two individual unused friction plates have been conducted in parallel. One plate was tested on a tribometer rig (heating in contact surface and wearing), while the other plate was dried in an oven (only heating, uniform one). Both plates were from the same production batch and exposed to the same temperature change schedule and generally to the same (plate storing) conditions during day-to-day and weekend pauses.

A procedure that compensates for the apparent moisture content influence when reconstructing the wear rate has been applied to the rig-tested friction plate data based on a correction drawn from the oven-dried plate data. It has been shown that the observed wear rate transient effect is suppressed after applying the proposed compensation procedure by approximately 50%; thus, indicating that the transient effect can indeed be predominantly explained by the moisture influence.

However, the moisture effect compensation approach is not practical for routine, long-time wear characterization tests, as it requires conducting synchronized tests involving two friction plates. Instead, it is recommended to solely reorganize the rig tests in the following manner, with the goal of suppressing the temperature-caused moisture content variation effect:

(i) design the experiments in the way that the operating points to be considered are grouped in sets of equal target temperature (other operating parameters should be randomized);
(ii) record a run-in set of experiments after a long pause or after the temperature operating point change, which would last until the transient wear effect ceases; and
(iii) introduce a ‘blind’ wear rate experiment at the beginning of each workday and set its target temperature to the level of the next, real test to heat up the pressure and friction plates and further mitigate the moisture effect.

Future research on the topic might include wear characterization using small samples of the friction materials and dedicated pin-on-disc rigs, which would have greater measurement precision and better control of ambient conditions, including air temperature and humidity. This activity could provide deeper insights into the physical background of the wear processes, as well as the influence of sample size and shape on the wear characterization results.

## Figures and Tables

**Table 1:** Cumulative difference in mass for rig-tested Plate C and oven-dried Plate D.

Nb.	Temp.	C	D	Nb.	Temp.	C	D	Nb.	Temp.	C	D
1	20	0.00%	0.00%	39	20	−2.75%	−1.14%	77	Low	−5.32%	−1.78%
2	100	−0.29%	−0.25%	40	100	−2.90%	−1.26%	78	20	−5.25%	−1.62%
3	100	−0.45%	−0.39%	41	Medium	−2.98%	−1.30%	79	100	−5.36%	−1.75%
4	100	−0.53%	−0.46%	42	Medium	−3.06%	−1.31%	80	Low	−5.41%	−1.80%
5	100	−0.59%	−0.50%	43	Medium	−3.14%	−1.30%	81	Low	−5.50%	−1.79%
6	100	−0.63%	−0.54%	44	Medium	−3.22%	−1.31%	82	High	−5.71%	−1.98%
7	20	−0.51%	−0.44%	45	Medium	−3.30%	−1.31%	83	High	−5.83%	−2.02%
8	100	−0.64%	−0.56%	46	20	−3.16%	−1.14%	84	20	−5.70%	−1.80%
9	100	−0.69%	−0.59%	47	100	−3.52%	−1.29%	85	100	−5.79%	−1.91%
10	100	−0.71%	−0.62%	48	Medium	−3.54%	−1.30%	86	High	−5.95%	−2.06%
11	Medium	−1.04%	−1.19%	49	Medium	−3.61%	−1.29%	87	High	−6.04%	−2.09%
12	Medium	−1.16%	−1.28%	50	Medium	−3.68%	−1.30%	88	High	−6.14%	−2.15%
13	Medium	−1.30%	−1.35%	51	High	−3.89%	−1.58%	89	High	−6.23%	−2.21%
14	Medium	−1.41%	−1.36%	52	High	−4.03%	−1.71%	90	High	−6.33%	−2.26%
15	Medium	−1.47%	−1.36%	53	20	−3.89%	−1.50%	91	High	−6.43%	−2.30%
16	20	−1.34%	−1.16%	54	100	−3.99%	−1.59%	92	20	−6.28%	−2.06%
17	100	−1.47%	−1.27%	55	High	−4.12%	−1.74%	93	100	−6.38%	−2.16%
18	Medium	−1.58%	−1.35%	56	High	−4.23%	−1.84%	94	High	−6.51%	−2.33%
19	Medium	−1.69%	−1.35%	57	High	−4.34%	−1.90%	95	High	−6.61%	−2.39%
20	Medium	−1.76%	−1.34%	58	High	−4.44%	−1.90%	96	Low	−6.66%	−2.30%
21	Medium	−1.91%	−1.33%	59	High	−4.54%	−1.91%	97	Low	−6.75%	−2.28%
22	20	−1.79%	−1.15%	60	Medium	−4.59%	−1.87%	98	20	−6.54%	−1.99%
23	100	−1.91%	−1.25%	61	20	−4.47%	−1.70%	99	100	−6.72%	−2.16%
24	Medium	−2.00%	−1.30%	62	100	−4.57%	−1.78%	100	100	−6.75%	−2.18%
25	Medium	−2.11%	−1.30%	63	Medium	−4.66%	−1.86%	101	Low	−6.79%	−2.27%
26	Low	−2.20%	−1.28%	64	Medium	−4.74%	−1.84%	102	Low	−6.87%	−2.27%
27	Low	−2.25%	−1.28%	65	Medium	−4.82%	−1.81%	103	20	−6.75%	−2.06%
28	20	−2.12%	−1.10%	66	Medium	−4.89%	−1.85%	104	100	−6.89%	−2.19%
29	100	−2.26%	−1.22%	67	20	−4.66%	−1.55%	105	Low	−6.93%	−2.26%
30	Low	−2.31%	−1.28%	68	100	−4.83%	−1.71%	106	Low	−7.01%	−2.24%
31	Low	−2.39%	−1.28%	69	100	−4.85%	−1.73%	107	Medium	−7.12%	−2.31%
32	Low	−2.46%	−1.27%	70	Medium	−4.91%	−1.84%	108	20	−6.97%	−2.07%
33	20	−2.28%	−1.01%	71	Medium	−4.99%	−1.85%	109	100	−7.10%	−2.19%
34	100	−2.46%	−1.14%	72	Low	−5.09%	−1.78%	110	Medium	−7.18%	−2.30%
35	Low	−2.54%	−1.22%	73	20	−4.97%	−1.61%	111	Medium	−7.26%	−2.31%
36	Low	−2.61%	−1.25%	74	100	−5.10%	−1.72%	112	Medium	−7.32%	−2.31%
37	Medium	−2.77%	−1.31%	75	Low	−5.17%	−1.77%	113	Medium	−7.39%	−2.30%
38	Medium	−2.88%	−1.31%	76	Low	−5.26%	−1.79%				

## Data Availability

No new data were created or analyzed in this study. Data sharing is not applicable to this article.
